# Effects of management strategies on animal welfare and productivity under heat stress: A synthesis

**DOI:** 10.3389/fvets.2023.1145610

**Published:** 2023-03-15

**Authors:** Joana Nazaré Morgado, Emilia Lamonaca, Fabio Gaetano Santeramo, Mariangela Caroprese, Marzia Albenzio, Maria Giovanna Ciliberti

**Affiliations:** ^1^Nutrition Laboratory, Environmental Health Institute, Faculty of Medicine of the University of Lisbon (FMUL), Lisboa, Portugal; ^2^Lisbon School of Economics and Management (ISEG), University of Lisbon, Lisboa, Portugal; ^3^Department of Agriculture, Food, Natural Resources, and Engineering (DAFNE), University of Foggia, Foggia, Italy

**Keywords:** animal welfare, biomarker, heat stress, livestock productivity, ruminants, THI

## Abstract

Climate change includes different dramatic events, and among them, heat stress exposition is the strongest phenomenon affecting the livestock sector. The effects of heat stress events on animal welfare are complex and the economic impacts for the livestock sector are relevant. Management measures may contribute to improve the resilience to heat stress, but the extent to which they impact on livestock performances and management strategies depend on the magnitude of the stress conditions. Through a pioneering synthesis of existing knowledge from experiments conducted in controlled conditions, we show that management strategies, both adaptation and mitigation measures, halved the negative impacts on the ruminants' performances and welfare induced by heat stress, but the efficacy is low in extreme conditions, which in turn are more and more frequent. These novel findings emphasize the need to deepen research on more effective adaptation and mitigation measures.

## 1. Introduction

A bilateral nexus links climate change (CC) and livestock. On one hand, livestock production processes are responsible for large environmental impacts driving CC. Agriculture is the second sector, after energy and industry, for levels of gate greenhouse gas (GHG) emissions. In particular, GHG emissions from rice and livestock products cover over 80–85% percent of total agricultural sector emissions, of which the majority comes from livestock (1). Specifically, ruminants contribute to 39.8% of world agricultural GHG emissions due to enteric fermentation (2).

Enteric fermentation and manure deposition on pastures dominates farm-gate GHG emissions, generating 3 billion tons of carbon dioxide equivalent (CO_2_eq) in 2018 (i.e., 32% of global emissions due to agriculture within the farm gate). Methane and nitrous oxide emissions from agricultural activities contributes to 5.3 billion tons CO_2_eq in 2018 and two-thirds of this total is attributed to livestock production. On the other hand, CC exerts a high pressure on livestock welfare. Experiments and observations have demonstrated that CC alters livestock welfare and farm profitability (3). Under CC influence, milk yields suffer reductions (4), feed intake, conception rates, feed supplies, carrying capacities, and grass nutrition are affected (5), and the spread of illness, disease and parasites' growth is favored (6). Furthermore, the increase of ambient temperature, higher than the upper critical temperature of a species' normal range, will be responsible for a worsening heat stress (HS) exposition in animals (7). Specifically, HS is defined as the sum of external forces acting on an animal, causing an increase in body temperature and evoking physiological responses (8). Depending on the level of intensity of a stress response, the activation and degree of stimulation of different systems (particularly immune system) have direct consequences on animal health and welfare conditions (9). During stress exposition, the central nervous and the immune systems are interconnected *via* the activation of the autonomic nervous system and the hypothalamic-pituitary-adrenal (HPA) axis through the secretion of neuropeptides and hormones, including glucocorticoids (10). In particular, at the initial phase of thermal exposure an increase in peripheral cortisol level is observed, to which a decrement follows when a prolonged exposure to hyperthermia occurs (11). Cortisol is mainly considered an immune suppressor, therefore higher level of cortisol further leads to the increase of immunosuppression and higher susceptibility to infectious diseases (12). Recently, a bidirectional effect of cortisol (immunosuppressive and anti-inflammatory) on the immune system in sheep depending on the magnitude of the stressor and the duration of the stress was defined (9).

Throughout the years, animals have developed a phenotypic response named heat acclimation, that includes reduction of feed intake, an increase of water intake, and an overall altered reproductive and productive efficiency together with other modified physiological functions (13, 14). In this context, the livestock sector needs to rely on management strategies which are both adaptation strategies facing pressures imposed by CC, and mitigation strategies limiting damages on the environment (15, 16). Particularly, the adaptation strategies include developing tolerant breeds, improving water access, enhancing pasture species, whereas the mitigation strategies cover nutritional interventions (e.g., adjustments in feeding including altered forages, nutrients' supplementation), manipulation of the rumen eco-system, provision of shade, housing, fans, and sprinklers (17–19).

While the nexus CC-livestock deserves attention on both directions, the present article focuses on the impacts of CC on ruminants' welfare and addresses the following research questions: (1) Which are the impacts of CC on ruminants' welfare? (2) Which is the role of management strategies in improving the resilience of the ruminants' sector to CC?

Recently, it has been preferred a holistic approach to understand the implications that CC events, particularly HS, have on livestock production, and the extent to which management strategies are effective is a way to protect not only the animals' health and welfare, but also the health of people and the environment, with a “One Health” approach (20). As set in the 27th United Nations Climate Change Conference –COP27– in November 2022, actions to address these interconnected issues and build resilience are needed with a matter of urgency, without much further ado (21). In line with this and with the United Nations' Sustainable Development Goals, management strategies can improve the resilience of crop and livestock productivity to CC.

Through a pioneering synthesis of existing knowledge, we investigated the impact of HS exposition on ruminants' welfare and performances and the contribution of management strategies. To determine the HS exposition levels, we used the Temperature Humidity Index (THI), a reliable environmental indicator which combines both temperature and relative humidity to measure thermal comfort or discomfort of animals (22). Particularly, THI has been used as useful tool to measure livestock productivity response as a function of climate (8, 23–26) and it is considered an indicative measure of “the sum of forces external to the animal that acts to displace body temperature from its set point” (8).

To the best of our knowledge, this is the first study which adopts THI variations to calculate CC impacts. The contribution of management strategies is assessed by comparing the effects both in thermoneutral vs. HS experimental conditions (i.e., unconditional of being in HS) and in HS experimental conditions only (i.e., conditional of being in HS).

## 2. Material and methods

### 2.1. Search strategy

A review of previous meta-analytical studies on the issue (27–35) allowed us to better define our research questions. These studies on the impacts of HS exposition on ruminants' welfare and performances were focused mainly on pigs and poultry. Our analysis complemented these studies by investigating both the impact of HS exposition and the contribution of management strategies conditional and unconditional of being in HS. Our study further expanded previous evidence by focusing on farmed ruminants, such as cattle, sheep, and goats, following the definition of ruminants in reference (36). The novelty of our study is in the use of the THI variations and of their magnitude to quantify the impacts of HS exposition on ruminants' performances and welfare. The impacts are evaluated for ruminants treated and non-treated with management strategies. We have conducted from the beginning of March until the end of April 2021 a systematic literature review on climate change effects on ruminants' welfare, following the guidance produced by the Preferred Reporting Items for Systematic Reviews and Meta-analyses (PRISMA) Statement (37). The completed PRISMA checklist is in the supporting information. The focus was on articles indexed in the Scopus database published until 2021. A combination of four groups of keywords have been used to select the articles of interest. Climate change-related terms (i.e., climate change, extreme weather, climatic stress, cold stress, heat stress, heat wave, thermo-hygrometric index, temperature humidity index, THI, and precipitation variation) allowed us to select articles focused on different climate change events. Animal welfare-related terms (i.e., animal welfare, immune response, immune-response, immunity, body condition score, BCS, body weight, respiration rate, conception rate, rectal temperature, milk yield, carcass weight, fat thickness, behavioral alteration, and behavioral alteration) identified articles analyzing animal-based welfare indicators, in order to portray the relevant influence that climate changes events have on the immune response and health of different species. Species-related terms (i.e., ruminant, small ruminant, small-ruminant, bovine, ovine, caprine, cattle, sheep, and goat) allowed to restrict the search to articles pertaining to the bovine, ovine and caprine species, and type of production (dairy and beef). Management strategy-related terms (i.e., management strategy, housing condition, cooling, ventilation, feeding strategy, antioxidant substance, antioxidant molecule, fat supplement, feed additive, amino acid, tannin, and circular economy) allowed us to identify the type of strategy used to ameliorate the CC effects on animals, with an emphasis on bedding type, nutritional (e.g., a special diet or supplement) and cooling (e.g., fans, sprinklers) management strategies.

### 2.2. Critical assessment of included studies

To be included in the sample, studies needed to meet the main following inclusion criteria: (a) papers under the category “Article,” that were published in the English language, for the sake of reproducibility, and in peer-reviewed journals, to exclude conference proceedings (38); (b) bovine, caprine, and ovine species were used as experimental animals; (c) for each experimental group, the THI had to be reported. The latter, depending on the study, may appear calculated using different formulas. Most of the literature based the THI calculation on the equation of Thom (39) [THI = 0.8 × T + ((RH/100) × (T−14.3)) +46.4], where T is the dry bulb air temperature (°C) and RH is the relative air humidity (%). Subsequently, the National Research Council (40) has given the algebraically equivalent equation: THI = (1.8 × T + 32) – (0.55 – 0.0055 × RH) × (1.8 × T – 26.8). Throughout the years a number of modifications and elaborations based on the basic THI equation have been proposed (41, 42). Concomitantly, several THI thresholds range have been published in the literature since different livestock species and other factors, such as geographic location (43). The thermoneutral thresholds are defined by physiological responses in relation to changes of animal's respiration rate and body temperature (44). In cattle, a THI of 74 or less is considered normal, 75–78 is alert status, 79–83 is danger status, and a THI equal to or above 84 is an emergency (39). More recently, in dairy cattle, the HS threshold has been set at daily THI values of 68–72 (45–47), although lower values have been already found for temperate areas (48–50). Moreover, in a recent assessment in dairy systems of the United States on the influence of variation in the THI on milk yields over 1981–2018, it was stated that both extreme heat (>79 THI) and cold (< 39 THI) impact milk yields with a reduction of daily yield from 3.7 to 6.1%, respectively, in comparison to the optimal conditions (65–69 THI) (51).

**Figure 1:**
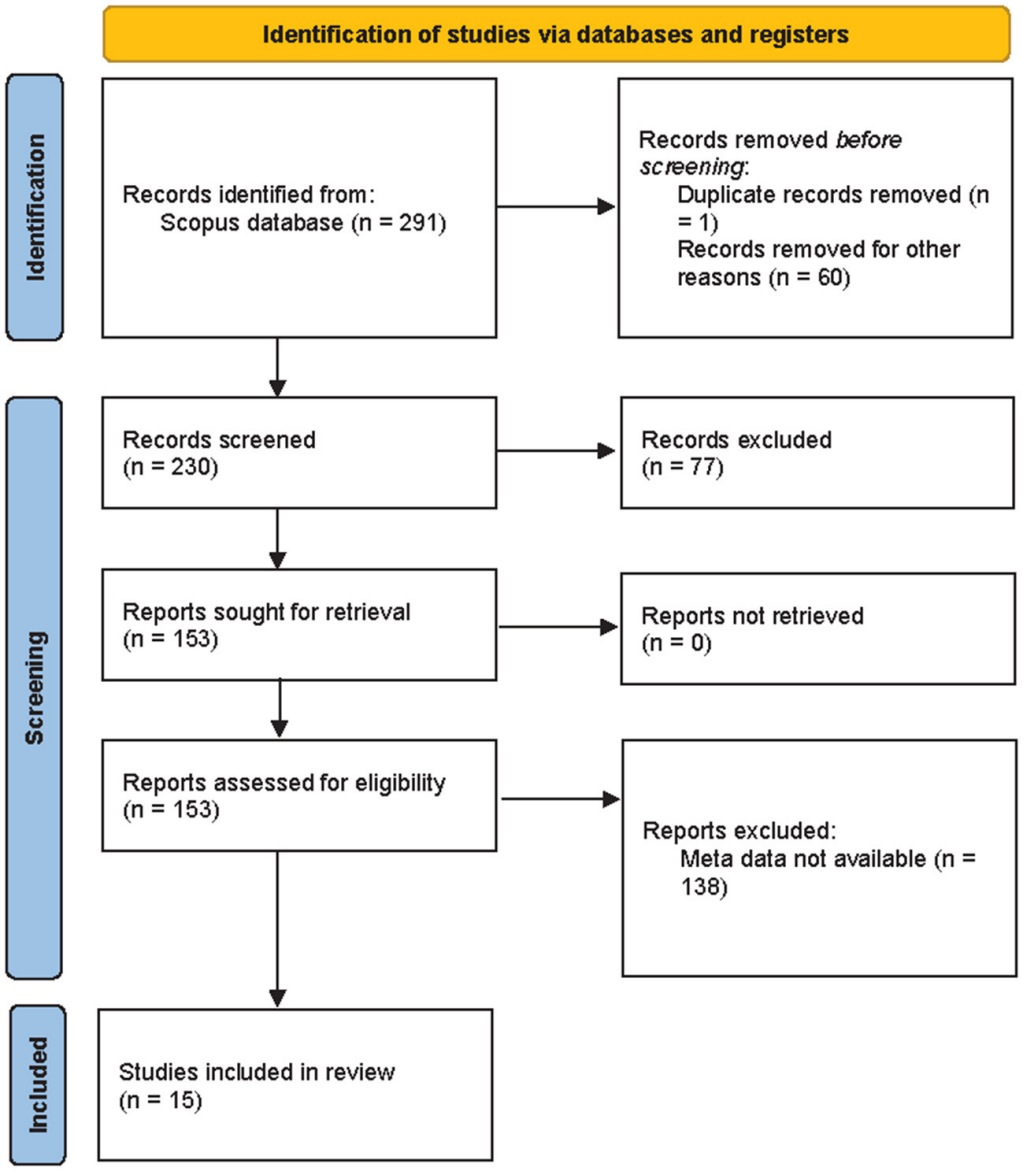
PRISMA 2020 flow diagram for the systematic review.

In sheep and goats, when the temperature is expressed in °C, the values obtained indicated the following: THI < 22.2 (absence of heat stress); THI from 22.2 to < 23.3 (moderate heat stress); THI from 23.3 to < 25.6 (severe heat stress) and THI ≥ 25.6 (extreme severe heat stress) (52). When temperature is expressed as °F the values obtained indicate the following: values < 82 = absence of heat stress; 82 to < 84 = moderate heat stress; 84 to < 86 = severe heat stress and over 86 = extreme severe heat stress (53). The systematic search, described in Figure 1, allowed to identify an initial set of 291 records containing in their title, abstract, or keywords all possible combinations of selected keywords. After removing records out of scope of this analysis (*n* = 60) and duplicates (*n* = 1), the selected records (*n* = 230) were screened based on information contained in titles, abstracts, and full texts. After excluding out of scope records (*n* = 77), 153 records were sought for retrieval and then assessed for eligibility. Three reviewers worked independently to screen each record and each report retrieved. After excluding records without meta-data suitable for the meta-regression analysis (*n* = 138), the final sample consisted of 15 article and 747 observations.

A critical assessment of included studies is provided in the Supplementary material (see Section “Description of the sample”). The high number of observations is due to the huge amount of information collected from the articles. For each article, each indicator of animal performances and welfare is considered as a separate entry in the dataset. Moreover, for articles describing more than one trial, each trial was considered as a separate one; and for studies comparing more than one group to a control group, each group was included separately. To that purpose, the inclusion criteria used were the following: (i) experiments had to analyze the animals' performance under both HS and thermoneutral (TN) conditions, (ii) or animals' performances under only under TN conditions; (iii) TN and HS groups had to encompass a management strategy to compare and observe the different effects; (iv) when considering the studies as in (ii) then criteria (iii) was not applicable. A detailed description of management strategies is provided in the Supplementary material (see Section “Management strategies description”).

### 2.3. Data extraction

We have extracted several data from each article. General information about the article, such as the year of publication, the journal in which the article has been published, the subject area in the Scopus database to which the journal belongs, the rank of the journal for each subject area at the date of publication, allowed to monitor the prestige of the articles. Information related to structural characteristics of the study, such as information on the countries of origin (e.g., Australia, China, Egypt, Germany, South Korea, Spain, and United States), species (e.g., bovine, caprine, and ovine), and type of production (e.g., dairy and growing or beef cattle, for milk and meat production, respectively), as well as breeds (i.e., Bedouin, Holstein-Friesian) have been gathered to account for geographical and animal-based differences in the relevance to CC and animal welfare. Information on methodology, such as methods used for statistical analyses (e.g., ANOVA, Mixed Models), information on the experimental design applied (e.g., randomized block, cross-over), data on experimental groups (i.e., THI reported either in TN and HS conditions, with or without mitigation strategy applied, time of detection of THI, and calculation formula used for THI). The degrees of HS for small ruminants (ovine and caprine) were classified as in references (52, 54). The heterogeneity of the THI equations and therefore the HS classification according to the THI threshold values for the different species are reported in Supplementary Table 4 (see Section “Review of temperature humidity index”). Information on indicators of animal performances (e.g., related to welfare and productivity) was reported. For instance, the body temperature indices (e.g., rectal temperature and respiration rate) and/ or the blood parameters [e.g., glucose, insulin, haptoglobin, cortisol, and immunoglobin G (IgG)], and/ or the milk production and composition (e.g., milk yield, protein yield, and fat milk content) and/or conception/pregnancy rate parameters were indicated. The full list of indicators is available in Supplementary Table 2 (see Section “Description of the sample”).

### 2.4. Data analysis

We have built three indices to investigate the changes in performances. A first index captured the impact of climate on animal performances and welfare, measured by the relative change in animal performances between thermoneutral (*TN*) and heat stress (*HS*) conditions for a group of animals not treated with management strategies (i.e., control group):


(1) $$\begin{array}{l} Climate\;impact=\frac{performance_{\left(control\right)}^{HS}-performance_{\left(control\right)}^{TN}}{performance_{\left(control\right)}^{TN}} \end{array}$$

A second index considered the relative change in animal performances and welfare between TN and HS conditions for a group of animals treated with a strategy that allows them to improve the resilience to HS (i.e., treatment group). This index described the joint impact of climate and management strategies on animal performances and welfare:


(2) $$\begin{array}{l} Climate-strategy\;impact=\\ \frac{performance_{\left(treatment\right)}^{HS}-performance_{\left(treatment\right)}^{TN}}{performance_{\left(treatment\right)}^{TN}} \end{array}$$

A third index measured the impact of management strategies on animal performances and welfare built as the relative change in performances between animals treated and non-treated with any management strategies in HS conditions:


(3) $$\begin{array}{l} Strategy\;impact=\frac{performance_{\left(treatment\right)}^{HS}-performance_{\left(control\right)}^{HS}}{performance_{\left(control\right)}^{HS}} \end{array}$$

The indices assumed negative values when a worsening in animal performances and welfare is observed and positive values when there is an improvement in animal performances. The indices are zero when animal performances do not significantly change. The frequency of changes in animal performances due to changes in climate conditions are reported in Table 1. Supplementary Table 1 describes the frequency of changes in animal performances due to changes in climate conditions by species. Supplementary Figure 1 shows the distributions of changes in performances and welfare due to changes climate conditions, with details on wort and better performances of animals.

**Table 1:** Frequency of changes in animal performances and welfare due to changes in climate conditions.

**Performance**		**Climate impact**	**Climate-strategy impact**	**Strategy impact**
Worst		32%	29%	16%
of which	Severe climate variation	19%	13%	29%
No change		63%	67%	51%
of which	Severe climate variation	46%	56%	48%
Better		5%	4%	33%
of which	Severe climate variation	17%	8%	9%

“Climate impact” is the relative change in performances and welfare of animals in control groups (i.e., without adaptation strategy) between thermoneutral and climatic stress conditions; “Climate-strategy impact” is the relative change in performances and welfare of animals in treatment groups (i.e., with adaptation strategy) between thermoneutral and climatic stress conditions; “Strategy impact” is the relative change in performances and welfare between animals in control (i.e., without adaptation strategy) and treatment (i.e., with adaptation strategy) groups in climatic stress conditions. For each change in performance (i.e., worst, no change, and better), the percentage of change due to a severe climate variation (a mild climate variation is the baseline) is reported.

**Figure 2:**
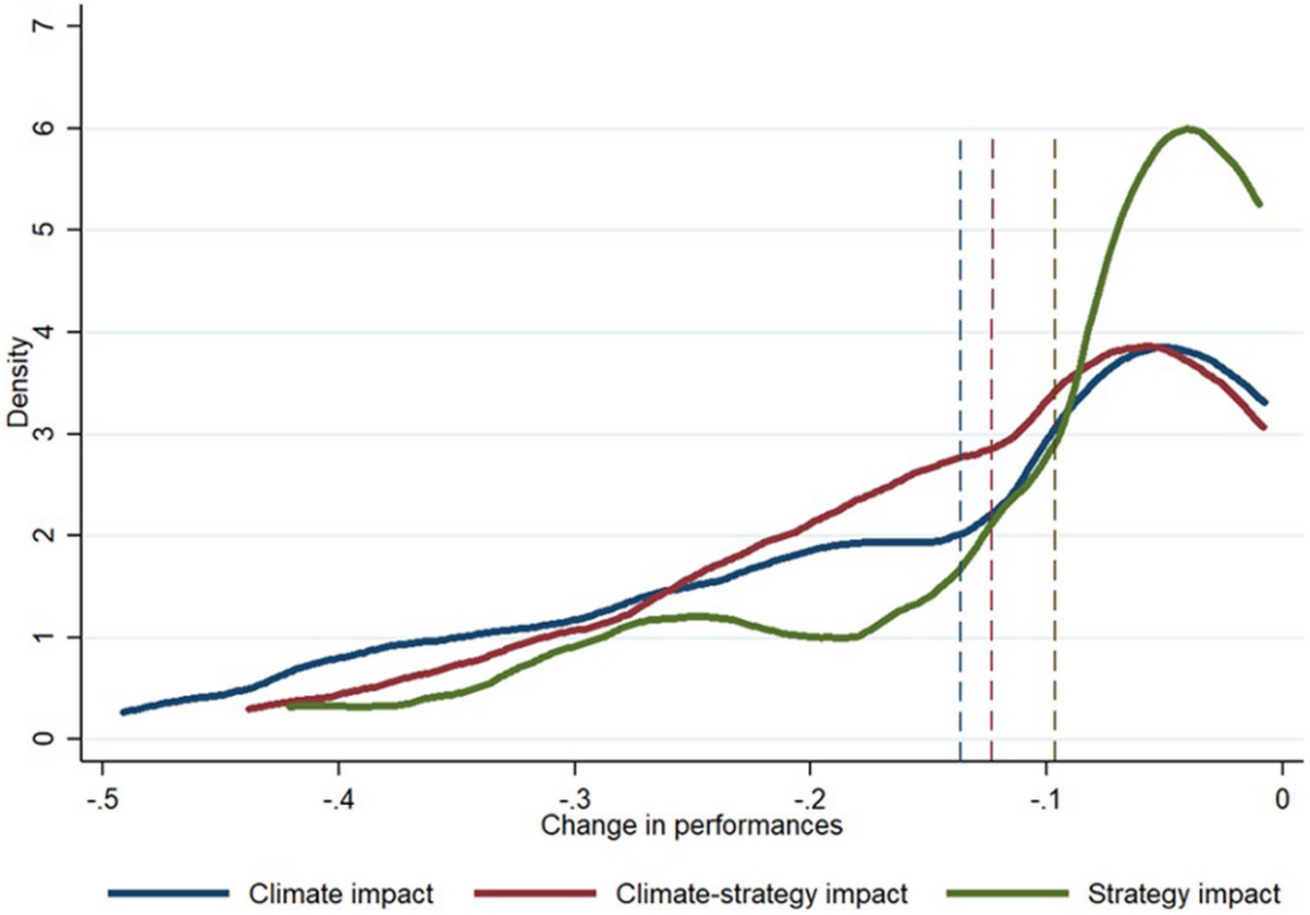
Distributions of changes in animal performances due to changes in climate conditions: details on worst performances.

From each index, we derived dummy variables that assume the value 1 if the indices are negative and 0 otherwise. The dummies indicated the frequency of worst performances. The average change in (worst) animal performances tend to approach to zero moving from “Climate impact” to “Strategy impact” highlighting the role of adaptation strategies in enhancing the resilience of animals to climate variations (Figure 2).

Distributions of indices include observations between 5 and 95th percentiles considering only worst performances. “Climate impact” is the relative change in performances and welfare of animals in control groups (i.e., without management strategy) between thermoneutral and heat stress conditions; “Climate-strategy impact” is the relative change in performances and welfare of animals in treatment groups (i.e., with management strategy) between thermoneutral and heat stress conditions; “Strategy impact” is the relative change in performances and welfare between animals in control (i.e., without management strategy) and treatment (i.e., with management strategy) groups in HS conditions. Dashed lines are the average change in animal performances (i.e., −0.143 for Climate impact, −0.129 for Climate-strategy impact, −0.097 for Strategy impact).

A meta-regression analysis was conducted to disentangle the effects of climate on animal performances. The model is as follows:


(4) $$y_{i}=\alpha +\beta X+\gamma THI+\Delta THI^{severe}+\varepsilon$$

The dependent variable (*y*_i_) is alternatively the dummies capturing the frequency of worst performances for each index, where *i* = *Climate impact; Climate*−*Strategy impact; Strategy impact* The climate variables of interest are Δ*THI* and Δ*THI*^*severe*^. Δ*THI* is the relative divergence of the THI in HS conditions with respect to TN conditions (i.e., $\frac{THI^{HS}-THI^{TN}}{THI^{TN}}$), Δ*THI*^*severe*^ considers severe divergences of the THI (i.e., Δ*THI* above the median): they both capture the effect of HS conditions on animal performances. γ and δ are the parameters of interest. When the model uses the dummy built on the index in equation (3), Δ*THI* is the THI in HS stress conditions normalized by the THI in TN conditions (i.e., $\frac{THI^{HS}}{THI^{TN}}$), Δ*THI*^*severe*^ considers severe THI in normalized HS stress conditions.

The model controls for structural characteristics of the articles included in the meta-analysis that are able to influence the heterogeneity observed in animal performances (55). In particular, we have explored the contribution of the characteristics of the experiment, such as the species and breeds used in the experiment, the countries in which the experiment is conducted, the type of strategy implemented to improve the resilience of animals to climate variations, the experimental design. Since these variables are highly correlated, a Principal Component Analysis (PCA) may help summarize this information. The two-thirds of the variance is contained in the first four principal components (Supplementary Figure 2): each of them accounts for more than 10% of the variance. The four components (Supplementary Table 4) are used as control factors *(X)* in the model in Equation 4.

As preliminary analysis, we estimated the model in Equation 4 through least squares using as dependent variable alternatively the indices in Equations 1–3 (cfr. results in Table 1). In a further analysis, we estimated Probit models using as dependent variable, alternatively, dummy variables build on indices in Equations 1–3 (cfr. results in Table 2). We also have analyzed the ability of the model to predict the contribution of climate conditions in explaining changes in worst performances (cfr. Table 3).

**Table 2:** Effects of heat stress conditions on performances.

**Variables**	**Climate impact**	**Climate-strategy impact**	**Strategy impact**
Delta THI	0.089	0.648	1.126^*^
	(0.357)	(0.612)	(0.647)
Severe delta THI	0.186	−1.871	−0.137^***^
	(0.187)	(1.493)	(0.052)
Observations	519	294	386

“Climate impact” is the relative change in performances and welfare of animals in control groups (i.e., without mitigation strategy) between thermoneutral and heat stress conditions; “Climate-strategy impact” is the relative change in performances and welfare of animals in treatment groups (i.e., with management strategy) between thermoneutral and heat stress conditions; “Strategy impact” is the relative change in performances and welfare between animals in control (i.e., without management strategy) and treatment (i.e., with management strategy) groups in heat stress conditions. Delta THI is the relative difference in average Temperature Humidity Index (THI) in thermoneutral and heat stress conditions. Severe delta THI considers the relative difference in average THI in thermoneutral and heat stress conditions above the median delta THI; in the specification “Strategy impact,” delta THI is the THI in heat stress conditions normalized by the THI in thermoneutral conditions. All specifications include a constant and the first four components derived from the Principal Component Analysis. Robust standard errors are in parentheses.

^***^Significant at the 10% level.

^*^Significant at the 10% level.

**Table 3:** Probability of observing worst performances and welfare due to heat stress conditions defined by THI.

**Variables**	**Climate impact**	**Climate-strategy impact**	**Strategy impact**
Delta THI	−1.091	−11.952^***^ [−3.149]	2.857
	(6.603)	(0.179)	(2.400)
Severe delta THI	−3.398	14.724^***^ [3.879]	−0.336
	(3.765)	(0.227)	(0.374)
Observations	519	294	386

“Climate impact” is the relative change in performances and welfare of animals in control groups (i.e., without management strategy) between thermoneutral and heat stress conditions; “Climate-strategy impact” is the relative change in performances and welfare of animals in treatment groups (i.e., with management strategy) between thermoneutral and heat stress conditions; “Strategy impact” is the relative change in performances and welfare between animals in control (i.e., without management strategy) and treatment (i.e., with management strategy) groups in heat stress conditions. Delta THI is the relative difference in average Temperature Humidity Index (THI) in thermoneutral and heat stress conditions; Severe delta THI considers relative difference in average THI in thermoneutral and heat stress conditions above the median delta THI; in the specification “Strategy impact,” delta THI is the THI in heat stress conditions normalized by the THI in thermoneutral conditions. All specifications include a constant and the first four components derived from the Principal Component Analysis. Robust standard errors are in parentheses. Average marginal effects are in brackets.

^***^Significant at the 1% level.

## 3. Results and discussion

Results from meta-regression analysis showed a non-linear relationship between animals' performances and HS conditions (“Strategy impact,” Table 2).

An increase in the THI in HS conditions is beneficial for the performances of animals treated with management strategies (positive coefficient estimated for “delta THI”), but only up to a certain point after which the animals' welfare and performances tend to worsen (negative coefficient estimated for “severe delta THI”). Our findings showed that the strategies adopted to improve the animals' performance and their resilience to HS played a mediator role in supporting animals' performances. However, this mechanism is less effective when the heat conditions are particularly severe. In a sensitivity analysis, we also examine which of the animal species is more affected by the impact of HS. The results, reported in the Supplementary Table 6, reveal that the detrimental effect of HS is almost attributed to caprine treated with management strategies. Caprine in our sample include Bedouin breed in Egypt and Murciano-Granadina raised in Spain, only the latter being exposed to severe HS conditions. This result can be explained by different physiology of stress activated by animals, and by type of management strategy applied in the samples. Goats are more thermo-labile than sheep, having different process of adaptation mechanisms (i.e., anatomical, morphological, physiological, feeding behavior, metabolism, and performance) (56). Among goat breeds, the different level of heat tolerance and thermoregulation response can be related to their morphological differences (57). As an example, animals with dark coat, characterized by greater absorption of thermal radiation, are found more susceptible to heat than those with light colored coat (58). Therefore, from our sample the management strategy applied to Murciano-Granadina, characterized by dark coat and exposed to severe HS, could be less effective due to more susceptibility of this breed.

The dynamic of HS response involves in well-orchestrated physiological processes that simultaneously influences multiple tissues and systems and is mediated by altered responses to homeostatic signals (59). Overall, small ruminants tend to be less susceptible to thermal stress than other domesticated ruminant species (19, 60), due to their unique genetic characteristics which improve the water conservation capability, the sweating rate, the respiration rate, the skin temperature, maintain a constant heart rate and constant cardiac output, and reduce the basal heat metabolism. All of previous characteristics enable these species to be more resilient (61) than bovine species, particularly high-producing dairy cows, which are the species with higher susceptibility to HS. It has been demonstrated that during HS exposition Murciano-Granadina goats show a reduction of feed intakes, normal blood glucose levels, and an absence of body fat mobilization due to a less sensitivity of adipose tissue to lipolytic signals (62). Similar to goats, sheep increase respiration rate, rectal temperature, alter the protein and energy metabolism, of mineral balance, enzymatic reactions, and hormonal secretions after the stimulation of temperature receptors located in the hypothalamus (63). Moreover, even if HS goats experienced negative energy balance, the level of circulating non esterified fatty acids (NEFA) are similar to goats in TN (62, 64, 65), which has been found also in dairy ewes (66), and cows (67). The absence of reduction in NEFA might be related to the essential role of insulin for the activation of the cellular stress response (68) mediated by the production of heat shock proteins aimed at protecting other proteins from heat-induced denaturalization (11). Recently, a downregulation of several anti-inflammatory pathways indicate that chronic HS goats have an inflammation status (69). In HS cattle an increase of >200-fold in heat shock protein 70 levels in blood lymphocytes are found (70). Thus, the synthesis of heat shock proteins contributes to the reduction of circulating aminoacids essential for milk protein synthesis (71). During HS exposition a condition of immune depression is ascertained in sheep (59, 63), in goats (72), and in cows (73, 74) explained as spare energy mechanism to reduce energetic costs related to cell growth and replication (67). Therefore, the alteration in immune responses can be strictly linked to the alterations activated in several tissues and systems to help the animals to cope with HS (59).

The relationship between likelihood of worst performances and HS conditions is also non-linear (Climate-strategy impact, Table 3).

When the relative divergence in THI, under TN and HS conditions, increases the likelihood of observing worst performances and welfare in groups of animals treated with any management strategy is 314.9% points lower under HS conditions: the strategies implemented to improve the animals' resilience toward HS are efficient. Nevertheless, these strategies have lower efficacy in case of severe HS variations. This is in line with Gonzalez-Rivas et al. (75), Gao et al. (76), Mehaba et al. (65), and Hamzaoui et al. (77). The likelihood of worst performances is 387.9% higher. The net effect is negative: if severe, HS variations increased by 73% the likelihood of worsening the animals' performances and welfare.

When animals are exposed to HS, a worsening of their performances and welfare is observed in about two-thirds of the cases (Table 4).

**Table 4:** Marginal contribution of heat stress conditions in explaining changes in worst performances and welfare.

	**Climate impact**	**Climate-strategy impact**	**Strategy impact**
**Prediction**			
${\hat{\gamma }}_{2}=\hat{\alpha }+\varepsilon$	32.18%	29.25%	16.32%
${\hat{\gamma }}_{2}=\hat{\alpha }+\hat{\beta }+\varepsilon$	26.97%	45.00%	21.04%
${\hat{\gamma }}_{2}=\hat{\alpha }+\hat{\beta }+\hat{\gamma }+\varepsilon$	26.33%	45.78%	22.50%
${\hat{\gamma }}_{3}=\hat{\alpha }+\hat{\beta }+\hat{\gamma }+\hat{\delta }+\varepsilon$	25.80%	56.03%	21.88%
**Marginal contribution**			
Delta THI $\left(\hat{\gamma }\right)$	−0.64%	+0.78%	+1.46%
Severe delta THI $\left(\hat{\delta }\right)$	−0.53%	+10.25%	−0.62%
Delta THI and severe delta THI $\text{(}\hat{\gamma }+\hat{\delta })$	−1.17%	+11.03%	+0.84%

“Climate impact” is the relative change in performances and welfare of animals in control groups (i.e., without management strategy) between thermoneutral and heat stress conditions; “Climate-strategy impact” is the relative change in performances and welfare of animals in treatment groups (i.e., with management strategy) between thermoneutral and heat stress conditions; “Strategy impact” is the relative change in performances and welfare between animals in control (i.e., without management strategy) and treatment (i.e., with management strategy) groups in heat stress conditions. Delta THI is the relative difference in average Temperature Humidity Index (THI) in thermoneutral and heat stress conditions; Severe delta THI considers relative difference in average THI in thermoneutral and heat stress conditions above the median delta THI; in the specification “Strategy impact,” delta THI is the THI in heat stress conditions normalized by the THI in thermoneutral conditions. Predictions are for the constant (α), the first four components derived from the Principal Component Analysis ($\hat{\beta }$), the delta THI $\left(\hat{\gamma }\right)$, the severe delta THI ($\hat{\delta }$). ε is the error term. The marginal contribution of $\left(\hat{\gamma }\right)$ is obtained by the difference ŷ_2_ – ŷ_1_, the marginal contribution of ($\hat{\delta }$) is obtained by the difference ŷ_3_ – ŷ_2_, the marginal contribution of ($\hat{\gamma }$ + $\hat{\delta }$) is obtained by the difference ŷ_3_ –ŷ_1_.

The occurrence of worst performances is lower of for animals treated with strategies improving their resilience (29.25%) than for animals without any type of management strategies (32.18%). This is consistent with the literature (15, 78). The management strategies improve animals' resilience, indeed, Rojas-Dowing et al. (15) suggested that a dietary strategy can reduce the risk from CC by increasing intake and protecting animals from malnutrition. The likelihood of worse animals' performances is (16.32%) lower for treated animals with respect to not treated animals (i.e., not subject to with management strategies).

Controlling for the experiments' conditions (i.e., ${\hat{\gamma }}_{1}$), we predict an increase in worst performances and welfare for treated animals. This is observed either when animals are exposed to HS (45.00%) and with respect to non-treated animals (21.04%). Similar effects are observed when controlling for HS conditions (i.e., ${\hat{\gamma }}_{2}$ and ${\hat{\gamma }}_{3}$). The worst performances and welfare of animals treated with management strategies increase by 0.78% due to HS and by 10.25% in case of severe HS. The severity of climate conditions tended to overcome the beneficial effect of management strategies. The HS increase by 1.46% the worst performances of treated animals with respect to non-treated animals. The strategies help protecting animals against severe HS (−0.62% of observing worst performances).

## 4. Conclusions

Two key points emerged from our analysis. First, we show that management strategies contribute to reduce the negative impacts of HS but are less effective when the HS is severe. Changes in weather conditions do not undermine welfare and performances of animals treated with management strategies. However, the effect of management strategies is less beneficial when changes in weather conditions are severe. Under severe climate conditions, even if the management strategies are applied, they did not help in restoring the stress condition which in turn involved in the perturbation of animal physiology and its health status. This could be due to a variety of factors that may affect ruminants' vulnerability to HS, such as: species, genetic potential, life stage, management, production, production system, and nutritional status (79). Adaptation is a function of the above-mentioned factors which are interrelated, and that either enhance or reduce adaptability. Even if a single thermal stressor may be important, the cumulative effects of multiple stressors (e.g., lactation stage, transition period, and dry period), in addition to that caused by HS exposition, may be significant and thus require further investigation. Second, we envisage the worsening of animal performances and welfare is likely to result in economic losses (80, 81). Although we have not quantified the losses, due to the impactable comparability of very diverse indicators, many of them are tightly connected to the economic performances (55).

In short, our analysis show that the HS exposition affected ruminants' performances and welfare. Regardless of being in HS (i.e., thermoneutral vs. HS experimental conditions), the management strategies reduce, by 3%, the negative impact on the performances.

Conditional of being in HS (i.e., in HS experimental conditions only), the occurrence of worst performances is 13% lower for animals treated with strategies than animals no treated with strategies.

Overall, the HS increase the probability of worst performances by 32%. Furthermore, with current adoptable strategies, the HS lower the performance as low as 16%: the (implied) efficacy of strategies is measured in about fifty percent.

Evidence from this analysis highlight the real urgency to discover new effectiveness strategies to help overcome the incoming more severe climate change. As suggested by Dunshea et al. (82) the combination of nutritional interventions with amelioration strategies, including housing and genetics, might be a more effective strategy to cope with HS challenge.

Further research is needed to improve the efficacy of management strategies. For instance, new technologies integration, such as precision feeding, and technology transfer systems potentially offer other opportunities for the future development of strategies.

## Data availability statement

The original contributions presented in the study are included in the article/Supplementary material, further inquiries can be directed to the corresponding authors.

## Author contributions

MCa and FS contributed to the conception and design of the study. MCa, FS, and MA supervised the overall project. JM, EL, and MCi organized the database and wrote the first draft of the manuscript. EL performed the statistical analysis. All authors contributed to manuscript revision, read, and approved the submitted version.
