# Supplementary material for: Effects of management strategies on animal welfare and productivity under heat stress: A synthesis
Source: Front Vet Sci. 2023 Mar 15;10:1145610. doi: 10.3389/fvets.2023.1145610 (PMC10050400; doi:10.3389/fvets.2023.1145610)
Supplement: Supplementary file 1 [file Data_Sheet_1.docx]

Supplementary Material

**Effects of management strategies on animal welfare and productivity under heat stress: a synthesis**

**Joana Nazaré Morgado^1,2^, Emilia Lamonaca^3*^, Fabio Gaetano Santeramo^3^, Mariangela Caroprese^3^, Marzia Albenzio^3^, Maria Giovanna Ciliberti^3*^**

^1^ Nutrition Laboratory, Environmental Health Institute, Faculty of Medicine of the University of Lisbon (FMUL), Lisboa, Portugal

^2^ Lisbon School of Economics and Management (ISEG), University of Lisbon, Lisboa, Portugal

^3^ Department of Agriculture, Food, Natural Resources, and Engineering (DAFNE), University of Foggia, Foggia, Italy

*** Correspondence:**

Corresponding Authors

[maria.ciliberti@unifg.it](mailto:maria.ciliberti@unifg.it), [emilia.lamonaca@unifg.it](mailto:emilia.lamonaca@unifg.it)

1. **Description of the sample**

Out of 231 articles retrieved from a Systematic Literature Review (SRL) on the Climate Change (CC)-ruminants nexus, 15 met the inclusion criteria for the quantitative analysis. More than two thirds of the articles have been published after 2015, a signal of the great awareness and the urgency of the recent years to better understand the CC effects on the livestock sector. The articles, published in high-ranked field journals indexed in Scopus, fall into the following subject areas: Agricultural and Biological Sciences (ABS), Biochemistry, Genetics and Molecular Biology (BGMB), Veterinary, and Multidisciplinary. Complementary, regarding the journals’ categories, the highest percentiles were under the ABS subject area, particularly for *Animal Science and Zoology*; and Multidisciplinary subject area, for *Multidisciplinary* category. The prestige of hosting journals contributes to improve the reliability of articles’ findings. Nevertheless, 3 out of the selected articles, were from journals that at the time of publishing appeared to be out of Scopus metrics’ coverage, which were the *Australian Journal of Agricultural Research* (2 articles, both published in 2004); and *Journal of Agriculture in the Tropics and Subtropics* (1 article, published in 1996). The first was discontinued from Scopus coverage in 2008 and followed into the subject area of Agricultural and Biological Sciences (category: General Agricultural and Biological Sciences; with percentile not stated); assuming from 2009 onwards the present name of *Crop and Pasture Science*, with subject area Agricultural and Biological Sciences, with two categories: Agronomy and Crop Science (73^rd^ percentile) and Plant Science (70^th^ percentile). The latter, *Journal of Agriculture in the Tropics and Subtropics* or *Tropenlandwirt*, is presently known as Journal of Agriculture and Rural Development in the Tropics and Subtropics, with Scopus coverage since 2002, and follows into two subject areas: Social Sciences (with two categories on Geography, Planning and Development, with 37^th^ percentile; and, Development, with 35^th^ percentile); and, Agricultural and Biological Sciences (with 3 different categories: Forestry, with 31^st^ percentile; Agronomy and Crop Science, with 28^th^ percentile; and, Animal Science and Zoology, with 26^th^ percentile). Despite data ranking on these journals having been discontinued from Scopus, the 3 articles were included not only due to the authors’ indices of citation (e.g., Gaughan, JB is cited in 5280 articles), but also due to the quality of the studies (e.g., protocol design, number, and type of health and welfare indicators evaluated, management strategy used); are considered relevant and eligible for our criteria.

Case studies of selected articles were conducted in Australia, the United States, Spain, China, Egypt, Germany, South Korea. This ensures a heterogeneous sample in terms of both starting climate conditions and heat stress (HS) events. As for the species, most of the studies were on bovine, followed by caprine and ovine. In 70 percent of articles on bovine species, the experiment was conducted on dairy cows (the remaining 30 percent is on beef cattle). The higher number of studies on dairy cows is motivated by findings from recent literature (Godyń et al., 2019), that refers dairy cows as those of concern for animal health and welfare.

In the sample, a worsening of animals’ performances and welfare due to HS conditions is observed in more than the half of cases when dairy cows are involved (Table S.1).

High-producing dairy cows, with expected high milk production performances, are prone to be more sensitive to thermal climatic stress and can be explained by the decrease in the temperature threshold when milk losses begin to increase, metabolic heat output is increased as production levels of the animal increase (Habeeb et al., 2018). On the other hand, beef cattle are considered to have less thermal sensitivity than dairy cows, since they have lower metabolic rate and lower body heat production; also, they tend to compensate for increased body temperature by igniting homeostatic mechanisms (higher levels of sweating, increased urination frequency), and altered behaviour (e.g., reducing activity and feed intake, and increased water intake) (Silanikove, 2000). Sheep and goats suffer less HS as compared to bovines, which enables them to survive in different regions and in some of the most inhospitable places of the world, and this is due to their unique characteristics such as water conservation capability, higher sweating rate, lower basal heat metabolism, higher respiration rate, higher skin temperature, constant heart rate, and constant cardiac output (Sevi and Caroprese, 2012). In particular, goats are considered capable for coping with multiple stressors better than sheep (Sejian et al., 2018).

We explored the contribution of different indicators of animal performances. The Figure S.1 shows the distributions of changes in performances and welfare due to changes climate conditions with details on wort and better performances of animals. The following Table S.2 shows the percent frequency of indicators in case of worsening of animal performances and welfare by indices.

When the external temperature reaches values below or above the thermoneutral zone (TNZ), animals have to actively adapt by playing out physiological, morphological and behavioural adaptation mechanisms, influencing differently the stress indicators of animal performances, health, and welfare (Baumgard and Rhoads, 2013). In our sample of articles, the different type of indicators found can be highlighted with the following main categories: feed and water intake parameters, e.g. feed intake (FI), dry matter intake (DMI) or daily dry matter intake (DMId), water intake; body temperature, e.g. Rectal Temperature (RT); Respiration Rate (RR), skin temperature (Ts); blood parameters, e.g. glucose, insulin, Blood Urea Nitrogen (BUN), Non-esterified Fatty Acids (NEFA), pH; milk production and composition, e.g. milk yield (MY), protein yield (PY), lactose yield (LY), fat yield (FY); reproduction, e.g. progesterone, oestradiol; immune response, e.g. cytokines, such as interleukins (IL-4, IL-6), Interferon γ (IFN-γ), and Tumour Necrosis Factor alpha (TNF-α).

The different indicators and the diverse response mechanisms that may explain the influence of HS on the species studied will be discussed particularly to dairy ruminants for the higher susceptibility to HS and economic relevance purpose. The decrease in daily feed intake (FI) is the first consequence that HS has on ruminants. HS negatively affects the hypothalamic appetite centre by reducing both FI (Baile and Forbes, 1974) and the heat production due to the animal’s metabolism (Kadzere et al., 2002; Speakman and Krol, 2010). Nevertheless, among other ruminants, goats are the ruminants that better resist to temperature variations (Bernabucci et al., 2010). In goats, despite the decrease in DMI, there is only a slight reduction in milk production (3–10%), with a minor degree of reduced contents of fat, protein, and lactose, compared to other dairy ruminants (Hamzaoui et al., 2010; Salama et al., 2014). The reduction of milk fat is not accompanied by a reduction in glucose levels or an increase of insulin levels as in cows (Hamzaoui et al., 2010; Salama et al., 2014). In lactating cows, the FI begins to decrease at an environmental temperature around 25–26 ^°^C and the reduction can reach the 40% when it reaches 40^°^C (Rhoads et al., 2013). Whereas at these temperatures dairy goats reduced by 22–35% the FI (Hamzaoui et al., 2010). Overall, the effect of HS on goats seems milder than in highly producing dairy cattle and the metabolic consequences may be attenuated with respect to those in cattle. In ruminants, body-core temperature indicators are widely used to indicate internal temperature during HS, particularly in cows (Hoffmann et al., 2013; Unruh et al., 2017). These indicators are mainly represented in all articles included in our study, as they are considered first indicators of thermal comfort, perceived as good primary care management practice when assessing health and welfare conditions of animals; they are relatively easy to measure and are part of the welfare quality protocol (Welfare Quality, 2009). Among all body-core temperature indicators, rectal temperature (RT) can be considered the best physiological indicator of HS (Silanikove, 2000). However, RT is not an easy trait to be routinely registered in a large population; therefore, other body-core temperatures (e.g., skin temperature, flank temperature) and respiration rate (RR) are considered the ideal complementary physiological indicators of animal’s overheat (Godyń et al., 2019; Pinto et al., 2020).

1. **Management strategies description**

Consistent with the inclusion criteria, all the articles investigate the animals’ performances and welfare under both thermoneutral (TN) and heat stress (HS) conditions. All but 3 articles encompass experiments that implement management strategies to compare and observe the different effects on performances of animals in both TN and HS groups. As for management strategies, most of the studies had a dietary (e.g., supplementation of zinc, propylene glycol, L-carnitine) and feed management (e.g., altered feeding time and/or feed consumption) strategies, followed by cooling management (e.g., sprinklers) and bed treatment strategies.

Adaptation strategies can improve the resilience of ruminants’ productivity to CC; whereas mitigation measures could significantly reduce the impact of ruminants on CC (Rojas-Downing et al., 2017). However, depending on the practices, livestock systems and location, literature may differ. On one hand, one study (Bernabucci, 2019) refers to adaptation strategies as of including production system adjustments and genetic improvement for thermotolerance, but also considered that, in addition to adaptation, mitigation strategies should also be addressed, and thus including these as the changes in animal management systems (e.g., nutritional interventions, cooling systems). On the other hand, other studies (Zhang et al., 2017; Rivera-Ferre et al., 2016) presented reviews considering that feeding strategies are both adaptation and mitigation strategies. As in Rivera-Ferre et al. (2016) and following the strategies described in this study, classified as having mitigation potential, all the strategies under the managerial category also have adaptation potential, and are suitable for at least two of the three (grazing, mixed and industrial) farming systems categories (Table S.3).

To expand this knowledge base, as in reference (Zhang et al., 2017), and considering that our analysis is focused on the HS effects on a set of performances and welfare with or without management strategies that, when implemented, should allow to reduce the negative effects of HS, this article considers adaptation options to have an economic orientation, as the focus of our analysis’ results.

Welfare and production performance of lactating livestock animals under high ambient temperatures can be sustained by feeding strategies, that may include feed management (changing feeding time and/or frequency) and dietary strategies (Renaudeau et al., 2012). The first strategy, as in reference (Holt et al., 2004), with the scope to determine the physiological effects of altered feeding time and/or feed consumption of feedlot cattle exposed to both TN and HS, the main findings showed that HS-related adverse effects could be alleviate by utilizing either a limit-feeding regimen or altering bunk management practices to prevent feed from being consumed several hours prior the hottest portion of the day. Additionally, in sheep this strategy can affect positively, e.g., feeding dairy sheep in the late afternoon reduced their heat production during the warmer hours of the day, when thermal balance through conduction and radiation mechanisms is less efficient, and sustained their cellular immune responses (Sevi et al., 2001). Secondly, we refer to dietary strategies, for these are known to help ruminants, in particular dairy animals, to cope with the negative effects of HS. A large number of positive effects on thermoregulatory, physiological, and immunological mechanisms have been found in literature due to dietary practices [Rojas-Downing et al., 2017; Caroprese et al., 2011; 2012 and 2014). In our study, several dietary strategies were observed [Hamzaoui et al., 2013; Gao et al., 2017; Gonzalez-Rivas et al., 2016; Kassube et al., 2017; Lamp et al., 2015; Lee et al., 2019; Mehaba et al., 2019; Opgenorth et al., 2021). In reference (Gao et al., 2017) it was investigated the HS effects on production, digestibility, rumen fermentation, and blood/urine nitrogen parameters relative to pair-fed in TN (PFTN) controls. Results showed that HS directly reduces milk protein concentration, due to the decrease in plasma amino-acids (AA) and increase in urine nitrogen parameters, combined with similar microbial crude protein (MCP) synthesis, suggest that increased extramammary AA consumption occurs during HS, and that decrease in milk protein observed during HS might be the result of a lack of available precursors supplied to the mammary gland. Therefore, HS has direct effects on protein metabolism and productivity, with decreased milk protein content and yield, and a reduction in DMI cannot explain solely this decline. Indeed, results also for dairy cows in references (Kassube et al. 2017; Lamp et al., 2015) are complementary to those findings. In the study of Kassube et al. (2017) it was attempted to determine that with an adequate supply/infusion of essential AA (EAA), milk and milk protein productions would be promoted and AA oxidation reduced, with reduced muscle protein degradation; however, the findings of this study did not improve milk production in HS cows, but, although not measure, results on plasma AA suggest that the treatment with both EAA (methionine and lysine) and branched-chain amino acids (ML+BCAA) reduced muscle catabolism in HS cows. The other study (Lamp et al., 2015), by comparing the macronutrient oxidation in different physiological stages (late-gestating versus early-lactating) dairy cows fed either *ad libitum* during high ambient temperatures or pair-fed at TN, demonstrated that HS cows extensively degraded tissue protein, with the increase of plasma urea, creatinine, and methyl histidine concentrations. These articles demonstrate that the study of AA’s metabolism should receive more attention to improve dietary recommendations, as well as the importance of considering in the future the different physiological and thus productive stages when testing nutritional strategies for attenuating impairments of health, welfare, and performance. On the other hand, more promising findings on dietary strategies were observed on bovine male cattle studies (Lee et al., 2019, Opgenorth et al., 2021). One study ((Lee et al., 2019) determined the effects of *Saccharomyces boulardii* CNCM-1079 (SB) as a feed additive on performance and other indicators in calves. It showed that calves exposed to HS receiving SB, compared to the control group (TN), showed higher DMI and lower water consumption, Tr, and cortisol level; therefore, live SB is useful in the livestock industry as an alternative to conventional medication that can be added to milk replacer in young calves in HS conditions. Another paper (Opgenorth et al., 2021), in which beef cattle were supplemented with different types of dietary zinc (Zn), demonstrated that Zn amino acid complex (ZA) ameliorated the heat load in HS steers, with benefits on thermal indices, intestinal architecture characteristics, and biomarkers of leaky gut, and thus may be a useful strategy to minimize negative effects of HS.

Furthermore, the one study on ovine species (Gonzalez-Rivas et al., 2016) observed that feeding corn grain plus forage (CD) may be a useful management strategy to help reduce digestive metabolic heat production, and thereby allowing ruminants more opportunity to dissipate excess heat. On caprine species, both studies found were related to feed supplementation in dairy goats, one paper with Propylene glycol (PG) (Hamzaoui et al., 2013) and another on L-Carnitine (CAR) (Mehaba et al., 2019). The first, although PG supplementation reduced body weight loss, caused milk fat depression (MFD), a multifactorial disorder, seen in dairy cows due to starch-enriched diets (LCI, 1970), that can induce potentially adverse effects on animal health and bring about important economic losses, with reduced milk fat production and milk quality. The second study, CAR had no lactational effects on performance, but was efficiently absorbed suggesting a certain level of rumen protection. This way, improving feeding practices as an adaptation measure could indirectly improve the efficiency of livestock production (Havlík et al., 2013). Regarding cooling management, the studies included were: one in caprine (Abdel-Samee, 1996) and another in bovine species (Gaughan et al., 2004). Despite in the study in caprine, the treatment of HS goats by chilled drinking water having significantly improved physiological and biochemical body parameters and reduced the effects of HS on thermal and water balances and organic functions, the bovine species study showed that inconsistent cooling regimens of HS animals (heifers) may increase the susceptibility of cattle to HS and elicit different physiological and metabolic responses. This way, accounting not only for the type of cooling systems but also for the frequency and duration the cooling regimens may help this strategy’s future efficacy. Several studies have been published on cooling management (Sevi et al., 2001; Davison et al., 2016) and these are for all species known to reduce CC effects, contributing among other to enhanced cellular immunity by sustaining the animals’ thermoregulatory mechanisms improving health and performance, and thus causing economic losses (Ferreira et al., 2016).

Finally, regarding bed treatment strategies, only one study was included according to the criteria to be selected in our analysis and it is on bovine dairy cows (Ortiz et al., 2015). Findings show that the interaction between a bed treatment and a cooling system, allows for better heat exchange to a certain bed material. In this sense, this study supports the hypothesis that future research interaction between different types of strategies may help the improvement of adaptation and mitigation practices.

1. **Supplementary Figures and Tables**


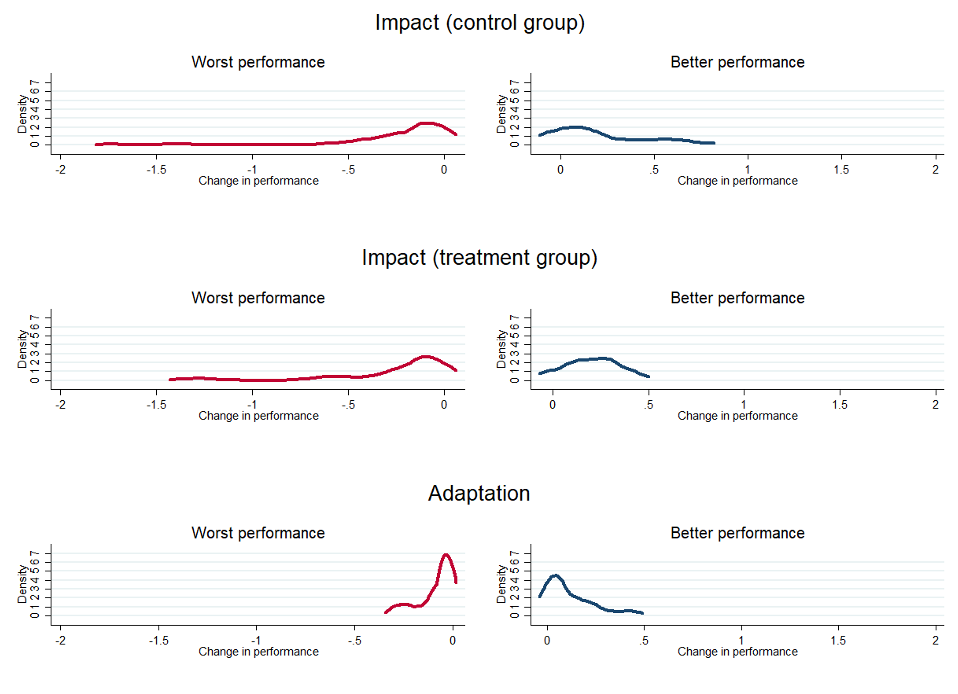


Distributions of negative change in performance and welfare (worst) and positive change in performance (better) include observations between 5^th^ and 95^th^ percentiles. ‘Impact (control group)’ is the relative change in performances and welfare of animals in control groups (i.e., without management strategy) between thermoneutral and climatic stress conditions; ‘Impact (treatment group)’ is the relative change in performances and welfare of animals in treatment groups (i.e., with management strategy) between thermoneutral and climatic stress conditions; ‘Adaptation’ is the relative change in performances and welfare between animals in control (i.e., without management strategy) and treatment (i.e., with management strategy) groups in climatic stress conditions. Dashed lines are the average change in animal performances and welfare (i.e., -0.143 for Climate impact, -0.129 for Climate-strategy impact, -0.097 for Strategy impact).

**Supplementary Figure 1.** Distributions of changes in performances and welfare due to changes climate conditions: details on wort and better performances.


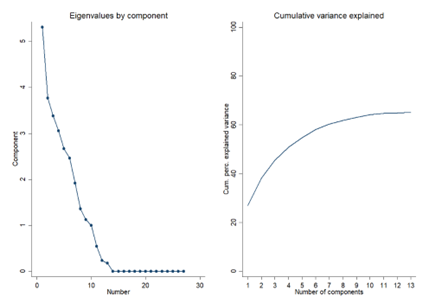


**Supplementary Figure 2.** Eigenvalues corresponding to each component and cumulative variation explained by each component.

**Supplementary Table 1.** Frequency of changes in performances and welfare due to changes in climate conditions.

|  |  |  | Climate impact | Climate-strategy impact | Strategy impact |
| --- | --- | --- | --- | --- | --- |
| Worst |  |  | 32% | 29% | 16% |
|  | of which |  |  |  |  |
|  | severe climate variation | | 19% | 13% | 29% |
|  | bovine |  | 56% | 100% | 81% |
|  |  | dairy cows | 58% | 78% | 71% |
|  | caprine |  | 33% | 0% | 3% |
| No change |  |  | 63% | 67% | 51% |
|  | of which |  |  |  |  |
|  | severe climate variation | | 46% | 56% | 48% |
|  | bovine |  | 78% | 67% | 61% |
|  |  | dairy cows | 83% | 84% | 66% |
|  | caprine |  | 22% | 33% | 37% |
| Better |  |  | 5% | 4% | 33% |
|  | of which |  |  |  |  |
|  | severe climate variation | | 17% | 8% | 9% |
|  | bovine |  | 74% | 100% | 59% |
|  |  | dairy cows | 82% | 42% | 61% |
|  | caprine |  | 9% | 0% | 33% |

‘Climate impact’ is the relative change in performances and welfare of animals in control groups (i.e., without adaptation strategy) between thermoneutral and climatic stress conditions; ‘Climate-strategy impact’ is the relative change in performances and welfare of animals in treatment groups (i.e., with adaptation strategy) between thermoneutral and climatic stress conditions; ‘Strategy impact’ is the relative change in performances and welfare between animals in control (i.e., without adaptation strategy) and treatment (i.e., with adaptation strategy) groups in climatic stress conditions. For each change in performance (i.e., worst, no change, better), the percentage of change due to a severe climate variation (a mild climate variation is the baseline) and type of bovine (beef cattle is the baseline) is reported.

**Supplementary Table 2.** Percent frequency of indicators contributing to worst performances and welfare due to climate conditions by indices.

| Indicators* | Climate impact | Climate-strategy impact | Strategy impact |
| --- | --- | --- | --- |
| Tr | 11.98 | 15.12 | 4.76 |
| RR | 10.78 | 12.79 | 4.76 |
| FI | 3.59 | 4.65 | 4.76 |
| Ts | 2.99 | 4.65 | 3.17 |
| MY | 2.4 | 4.65 | 3.17 |
| t_rest | 2.4 | 4.65 | 3.17 |
| CBT | 2.4 | 4.65 |  |
| Tbed_surf | 2.4 | 4.65 |  |
| Hr | 1.8 | 3.49 |  |
| Cortisol | 1.8 | 2.33 |  |
| Diar | 1.8 | 2.33 |  |
| Lact | 1.8 | 1.16 | 4.76 |
| Hct | 1.8 |  | 6.35 |
| Glucose | 1.8 |  | 4.76 |
| GCA | 1.8 |  | 1.59 |
| Hb | 1.8 |  | 1.59 |
| pCO_2_ | 1.8 |  |  |
| w_int | 1.8 |  |  |
| Tbed | 1.2 | 4.65 |  |
| PR | 1.2 | 2.33 |  |
| Fat | 1.2 | 1.16 | 1.59 |
| cHb | 1.2 |  | 3.17 |
| cTCO_2_ | 1.2 |  | 3.17 |
| cHCO_3_^−^ | 1.2 |  | 1.59 |
| pO_2_ | 1.2 |  | 1.59 |
| A | 1.2 |  |  |
| Cholest | 1.2 |  |  |
| DBG | 1.2 |  |  |
| DMId | 1.2 |  |  |
| DSG | 1.2 |  |  |
| G | 1.2 |  |  |
| Na^+^ | 1.2 |  |  |
| PCV | 1.2 |  |  |
| RBCs | 1.2 |  |  |
| SGPT | 1.2 |  |  |
| T_3_ | 1.2 |  |  |
| TBS | 1.2 |  |  |
| TBW | 1.2 |  |  |
| Ur | 1.2 |  |  |
| cSO_2_ | 1.2 |  |  |
| feed_ef | 1.2 |  |  |
| lipids_t | 1.2 |  |  |
| protein_t | 1.2 |  |  |
| w_int2 | 1.2 |  |  |
| Arg | 0.6 | 2.33 | 1.59 |
| Trp | 0.6 | 1.16 | 1.59 |
| Tv | 0.6 | 1.16 | 1.59 |
| MUN | 0.6 | 1.16 |  |
| Protein | 0.6 | 1.16 |  |
| heat_flux | 0.6 | 1.16 |  |
| DMI | 0.6 |  | 3.17 |
| K | 0.6 |  | 1.59 |
| NEFA | 0.6 |  | 1.59 |
| SAA | 0.6 |  | 1.59 |
| BUN ^53^ | 0.6 |  |  |
| FCM | 0.6 |  |  |
| H:D | 0.6 |  |  |
| HCO_3_^-^ | 0.6 |  |  |
| K^+^ | 0.6 |  |  |
| Na | 0.6 |  |  |
| TCO_2_ | 0.6 |  |  |
| Bwf | 0.6 |  |  |
| Jejheight | 0.6 |  |  |
| wc |  | 3.49 |  |
| Lys |  | 2.33 | 1.59 |
| Asn |  | 1.16 | 1.59 |
| Citrulline |  | 1.16 | 1.59 |
| Ornithine |  | 1.16 | 1.59 |
| Tyr |  | 1.16 | 1.59 |
| MSNF |  | 1.16 |  |
| NH3-Npf |  | 1.16 |  |
| VFAt |  | 1.16 |  |
| Butyrate |  | 1.16 |  |
| Ile |  | 1.16 |  |
| Ser |  | 1.16 |  |
| Thr |  | 1.16 |  |
| Crt |  |  | 6.35 |
| Sacchar |  |  | 4.76 |
| FY |  |  | 3.17 |
| LY |  |  | 3.17 |
| PY |  |  | 3.17 |
| Blood pH |  |  | 1.59 |
| bw |  |  | 1.59 |
| methyl_hist |  |  | 1.59 |

‘Climate impact’ is the relative change in performances and welfare of animals in control groups (i.e., without management strategy) between thermoneutral and climatic stress conditions; ‘Climate-strategy impact’ is the relative change in performances and welfare of animals in treatment groups (i.e., with management strategy) between thermoneutral and climatic stress conditions; ‘Strategy impact’ is the relative change in performances and welfare between animals in control (i.e., without management strategy) and treatment (i.e., with management strategy) groups in climatic stress conditions. ^*^Tr=rectal temperature; RR=Respiration rate; FI=feed intake; Ts=skin temperature; MY=reported milk yield for the treatment; t_rest=resting time; CBT=mean 24-h core body temperature; Tbed_surf=bed surface temperature; Hr= Heart rate; diar=Occurrence of diarrhea; Lact=lactose; Hct=Hematocrit; GCA=Goblet cell area, expressed as a percentage of epithelial area; Hb=Hemoglobin; pCO_2_=partial pressure of carbon dioxide (CO_2_); w_int=water intake; Tbed=bed temperature; PR=pulse rate; Fat=Fat milk content or fat; cHb=calculated value of hemoglobin; cTCO_2_=calculated value of total CO_2_; cHCO_3_^-^=calculated value of bicarbonate; pO_2_=partial pressure of oxigen (O2); A=Albumin; Cholest=Cholesterol; DBG=Daily body gain; DMId=Daily dry matter intake; DSG=Daily solids gain; G=Globulin; Na^+^=Ion Sodium; PCV=packed cell volume; RBCs=red blood cells; SGPT=glutamic pyruvic transaminase; T_3_=Plasma triiodothyronine; TBS=total body solids; TBW= total body water; Ur=Urea; cSO_2_=calculated value of oxygen saturation; feed_ef=Feed efficiency; lipids_t=Total lipids; protein_t=Total protein; w_int2=water intake in mL/kg of live body weight (LBW)/day; Arg=Arginine; Trp=Tryptophan; Tv=vaginal temperature; MUN=milk urea nitrogen; Protein=Protein milk content or True Protein; heat_flux=Heat flux; DMI=dry matter intake; K=Ion Potassium (blood); NEFA=nonesterified fatty acids; SAA=Serum amyloid A; BUN=Blood urea N; FCM=Fat-corrected milk (fat-corrected milk = kg of milk yield × [0.432 + 0.162 × (fat%)]); H:D=Villus height:crypt depth; HCO_3_^-^=hydrogencarbonate ion; TCO_2_=Total Carbon Dioxide (CO_2_); Bwf=Final body weight; Jejheight=Jejunum Height; wc=water consumption; Lys=Lysine; Asn=Asparagine; Tyr=Tyrosine; MSNF= milk solids non-fat; NH3-Npf=NH3-N prior feeding; VFAt: total volatile fatty acids; Ile=Isoleucine; Ser=Serine; Thr=Threonine; Crt=Creatinine; Sacchar=Saccharomyces spp.; FY=Fat yield; LY= Lactose yield; PY=Protein yield; bw=body weight; methyl_hist=1-/3-methyl histidine.

**Supplementary Table 3.** Qualitative integrated assessment of management strategies divided for the adaptation and mitigation strategies, and for the managerial category, and its potential applicability by livestock farming system, including the type of knowledge associated to develop the strategies (adapted from Rivera-Ferre et al. [2016]).

| **Category** | **Sub-category** | **Practices** | **Potential applicability** | | | |
| --- | --- | --- | --- | --- | --- | --- |
|  |  |  | **Mixed system** | **Industrial system** | **Co-benefits** | **Knowledge type** |
|  |  | **MITIGATION** |  |  | **ADAPTATION** |  |
| **Managerial** | Livestock nutrient cycling | Changes in feed quality and composition  (e.g., dietary strategy) | ++ | ++ | ++ | LTK/STK |
|  |  | **ADAPTATION** |  |  | **MITIGATION** |  |
| **Managerial** | Farm management | Altering timing of farming practices  (e.g., feed management) | ++ | 0 | 0 | LTK |
|  |  | Cooling system (e.g., sprinklers) | 0 | ++ | - | STK |

Potential applicability refers to relevance for the specific farming system, or capacity of the system to adopt such a strategy (e.g., poor farmers cannot adopt some expensive technologies). 0 can indicate a lack of potential or that the strategy is already part of the system (e.g., mixed livestock systems already integrate livestock and crops). + and ++ indicate greater degrees of potential for application. Knowledge type=LTK, Local and traditional knowledge; STK, Scientific and technological knowledge.

**Supplementary Table 4.** Review of temperature humidity index. Selected studies with the average temperature humidity index (THI) in Heat Stress conditions (HS) reported in our SLR included studies and the equation used to quantify the THI, with the respective HS classification**.**

| **Species – sex + type of production** | **Average THI in HS conditions** | **Onset of HS level**  **(THI threshold values used to classify HS level)** | | **HS level**  **(comments and/or references)** | **THI equation** | **SLR inclusion criteria**  **(category 1 or 1 and 2)** | **Study** |
| --- | --- | --- | --- | --- | --- | --- | --- |
| Bovine – female + dairy cows | 70.6 | Moderate HS | | The changes in core body temperature and respiration rate indicate that HS conditioning promoted moderate HS in the study’s experimental cows | Dikmen and Hansen , 2009 | Categories 1 and 2 | Kassube et al., 2017 |
| Bovine – female + dairy cows | 78.6 | THI level above 68 represents mild to moderate HS |  | | THI=Tdb^1^-(0.55-(0.55 × RH/100) × (Tdb-58)  (Tdb: dry bulb temperature (°C), Tdp: dew point temperature (°C))  (Zimbelman et al. 2009) | Categories 1 and 2 | Ortiz et al., 2015 |
| Bovine – female heifers | 82.1 | THI between 79 and 83 considered dangerous for ruminants | LCI [43] | | THI = (0.8 × Tdb) + [(RH/100)  × (Tdb − 14.4)] + 46.4 | Categories 1 and 2 | Gaughan et al., 2004 |
| Bovine – dairy | 84.5 | Severe/extreme HS level | cows in this study were exposed to a THI threshold value well above 68, established by Zimbleman et al. (2009) as the value from which milk production starts to decline due to HS conditions) | | THI=Tdb-(0.55-(0.55×RH/100) × (Tdb-58)  (Zimbelman et al., 2009]) | Categories 1 and 2 | Gao et al., 2017 |
| Bovine – dairy | 76.1 | Mild HS | THI between 72 to 79 considered as mild HS (Armstrong [44]). However, THI ≥75 will cause drastic decreases in production performance (De Rensis et al., 2015) | | THI = (1,8 × AT (°C) +32) - (0,55 - 0,0055 x RH (°C) × (1,8 x AT (°C) - 26)  (AT: ambient air temperature; RH: Relative humidity) | Categories 1 and 2 | Lamp et al., 2015 |
| Bovine – dairy | 83.2 | Moderate HS | THI between 80 to 90 considered as moderate HS (Armstrong, 1994). However, THI ≥75 will cause drastic decreases in production performance (De Rensis et al., 2015) | | THI = (0.8 x Ta) + ((RH / 100) x (Ta– 14.4)) + 46.4; Ta: ambient temperature; RH: relative humidity | Category 1 | Rispoli et al., 2019 |
| Bovine – dairy | 79 | Mild HS | THI between 72 to 79 considered as mild HS (Armstrong, 1994). However, THI ≥75 will cause drastic decreases in production performance (De Rensis et al., 2015) | | THI = Tdb + (0,36 × Tdp) + 41,2;  where Tdp = (237,3 × b)/(1,0 - b); b = [log(RH/100,0) + (17,27 ×Tdb)/ (237,3 + Tdb)] / 17,27 | Category 1 | Garner et al., 2017 |
| Bovine – beef | 74.5 | Moderate HS level | When the study does not refer to the HS classification, we use that from the meta-analysis in reference (Thornton et al., 2022) | | THI = (1,8 x T+ 32) - [(0,55 - 0,0055 × RH) x (1.8 × T - 26)] | Categories 1 and 2 | Opgenorth et al., 2021 |
| Bovine – beef | 82.9 | High HS level | When the study does not refer to the HS classification, we use that from the meta-analysis in reference (Thornton et al., 2022) | | THI = 0.8 × AT + [RH × (AT − 14.4)] + 46.4 (AT: ambient air temperature; RH: Relative humidity) | Categories 1 and 2 | Lee et al., 2019. |
| Bovine – beef | 70.7 | Mild HS (LCI, 1970) | HS classification from LCI (1970). According to the Holt et al. (2004): under hot conditions, mean THI exceeded 75 over 7h, which is well within the range of environmental conditions associated with Bos taurus cattle experiencing HS | | THI = (0.8 × Tdb) + [(RH/100)  × (Tdb − 14.4)] + 46.4 | Categories 1 and 2 | Holt et al. 2004 |
| Caprine – dairy | 82 | Between moderate and severe HS | Hamzaoui et al., 2013 | | THI = (1.8 × Tdb + 32) − [(0.55 − 0.0055 × RH) × (1.8 × Tdb − 26.8)] (Tdb: dry bulb temperature (°C), Tdp: dew point temperature (°C)) | Categories 1 and 2 | Hamzaoui et al., 2013 |
| ,Caprine – dairy | 79.5 | Moderate HS | (Hamzaoui et al., 2013) | | THI = (1.8 × Tdb + 32) − [(0.55 − 0.0055 × RH) × (1.8 × Tdb − 26.8)] (Tdb: dry bulb temperature (°C), Tdp: dew point temperature (°C)) | Categories 1 and 2 | Mehaba et al., 2019 |
| Caprine – dairy | 79 | Moderate HS | (Hamzaoui et al., 2013) | | THI = (1.8 × T + 32) − (0.55 − 0.0055 × RH) × (1.8 × T − 26), T: temperature (°C) and RH: relative humidity (%) | Category 1 | Contreras-Jodar et al., 2019 |
| Caprine – meat | 82.5 | THI over 78 is considered extreme distress | Livestock and Poultry Heat Stress indices suggested by Agricultural Engineering Technology Guide, Clemson University, Clemson, SC. 29634, USA (LPHSI,1990) | | THI = db − (0,55 − 0,55 RH) × (db − 58); db is the dry bulb temperature (°F) and RH is the relative humidity (%/100) | Categories 1 and 2 | Abdel-Samee, 1996 |
| Ovine – meat | 29.5 | Extreme severe HS | When values are in °F HS can be classified as follows: <82 = absence of HS, 82- <84 = moderate HS,  84-<86 = severe HS and  over 86 = very severe HS (Marai et al., 2001)  However, when the temperature is expressed in °C, the equation changes as follows:  THI = db *C – [(0.31 − 0.31 RH)(db °C − 14.4)], where db °C is the dry bulb temperature (°C) and RH is the relative humidity (RH%)/100. The values obtained indicate the following: THI < 22.2 (absence of heat stress); THI from 22.2 to <23.3 (moderate heat stress); THI from 23.3 to <25.6 (severe heat stress) and; THI ≥ 25.6 (extreme severe heat stress) (Marai et al., 2007). | | THI = db°C − [(0.31 − 0.31 RH/100) × (db°C − 14.4)]; db°C is the dry bulb temperature (°C) and RH is the relative humidity | Categories 1 and 2 | Gonzalez-Rivas et al., 2016 |

**Supplementary Table 5.** Principal components/correlation.

| Component | Eigenvalue | Difference | Proportion | Cumulative |
| --- | --- | --- | --- | --- |
| Comp1 | 5.312 | 1.541 | 0.197 | 0.197 |
| Comp2 | 3.771 | 0.390 | 0.140 | 0.336 |
| Comp3 | 3.381 | 0.328 | 0.125 | 0.462 |
| Comp4 | 3.053 | 0.389 | 0.113 | 0.575 |
| Comp5 | 2.664 | 0.200 | 0.099 | 0.673 |
| Comp6 | 2.464 | 0.544 | 0.091 | 0.765 |
| Comp7 | 1.920 | 0.563 | 0.071 | 0.836 |
| Comp8 | 1.357 | 0.232 | 0.050 | 0.886 |
| Comp9 | 1.125 | 0.128 | 0.042 | 0.928 |
| Comp10 | 0.997 | 0.455 | 0.037 | 0.965 |
| Comp11 | 0.541 | 0.303 | 0.020 | 0.985 |
| Comp12 | 0.238 | 0.062 | 0.009 | 0.994 |
| Comp13 | 0.176 | 0.176 | 0.007 | 1.000 |
| Comp14 | 0.000 | 0.000 | 0.000 | 1.000 |
| Comp15 | 0.000 | 0.000 | 0.000 | 1.000 |
| Comp16 | 0.000 | 0.000 | 0.000 | 1.000 |
| Comp17 | 0.000 | 0.000 | 0.000 | 1.000 |
| Comp18 | 0.000 | 0.000 | 0.000 | 1.000 |
| Comp19 | 0.000 | 0.000 | 0.000 | 1.000 |
| Comp20 | 0.000 | 0.000 | 0.000 | 1.000 |
| Comp21 | 0.000 | 0.000 | 0.000 | 1.000 |
| Comp22 | 0.000 | 0.000 | 0.000 | 1.000 |
| Comp23 | 0.000 | 0.000 | 0.000 | 1.000 |
| Comp24 | 0.000 | 0.000 | 0.000 | 1.000 |
| Comp25 | 0.000 | 0.000 | 0.000 | 1.000 |
| Comp26 | 0.000 | 0.000 | 0.000 | 1.000 |
| Comp27 | 0.000 | . | 0.000 | 1.000 |

**Supplementary Table 6.** Effects of heat stress conditions on performances by species.

| Variables | Climate impact | Climate-strategy impact | Strategy impact |
| --- | --- | --- | --- |
| Delta THI | 0.1147 | 0.2358 | 0.7750 |
|  | (0.2299) | (0.4973) | (0.5632) |
| Severe delta THI (bovine) | 0.1356 | -1.3620 | -0.011 |
|  | (0.1841) | (1.0922) | (0.0193) |
| Severe delta THI (caprine) | 0.3263 | -0.2358 | -0.2741** |
|  | (0.5686) | (0.4973) | (0.1326) |
| Observations | 519 | 294 | 386 |

**References**

Abdel-Samee, A. M. (1996). Heat adaptability of growing bedouin goats in Egypt, Journal of Agriculture in the Tropics and Subtropics. 96,137-147.

Armstrong, D. (1994). Heat stress interaction with shade and cooling. J. Dairy Sci. 1994; 77(7): 2044-2050.

Baile, C. A., & Forbes, J. M. (1974). Control of feed intake and regulation of energy balance in ruminants. Physiol Rev. 54, 160–214. doi: 10.1152/physrev.1974.54.1.160.

Baumgard, L. H., & Rhoads, R.P. (2013). Effects of heat stress on postabsorptive metabolism and energetics. annual review of animal biosciences. 1(1), 311–337. doi: 10.1146/annurev-animal-031412-103644.

Bernabucci, U. (2019). Climate change: impact on livestock and how can we adapt. Anim. Front: the review magazine of animal agriculture. 9(1): 3.

Bernabucci, U., Lacetera, N., Baumgard, L. H., Rhoads, R. P., Ronchi, B., Nardone, A. (2010). Metabolic and hormonal acclimation to heat stress in domesticated ruminants. Animal. 4, 1167–83. doi: 10.1017/S175173111000090X.

Caroprese, M., Albenzio M., Bruno, A., Annichiarico, G., Marino, R., Sevi. A. (2012). Effects of shade and flaxseed supplementation on welfare of lactating ewes under high ambient temperatures. Small Rumin Res. 102, 177–185.

Caroprese, M., Albenzio, M., Bruno, A., Fedele, V., Santillo, A., Sevi. A. (2011). Effect of solar radiation and flaxseed supplementation on milk production and fatty acid profile of lactating ewes under high ambient temperature. J Dairy Sci. 94, 3856–3867.

Caroprese, M., Ciliberti, M. G., Annicchiarico, G., Albenzio, M., Muscio, A., Sevi. A. (2014). Hypothalamic-pituitary-adrenal axis activation and immune regulation in heat-stressed sheep after supplementation with polyunsaturated fatty acids. J Dairy Sci. 97, 4247–4258.

Contreras-Jodar, A., Nayan, N. H., Hamzaoui, S., Caja, G., Salama. A. A. (2019). Heat stress modifies the lactational performances and the urinary metabolomic profile related to gastrointestinal microbiota of dairy goats. PLoS One. 14(2), e0202457.

Davison, T. M., Jonsson, N. N., Mayer, D. G., Gaughan, J. B., Ehrlich, W. K., McGowan. M. R. (2016). Comparison of the impact of six heat-load management strategies on thermal responses and milk production of feed-pad and pasture fed dairy cows in a subtropical environment. Int. J. Biometeorol. 60(12),1961–1968. doi: 10.1007/s00484-016-1183-2.

De Rensis, F., Garcia-Ispierto, I., López-Gatius, F. (2015). Seasonal heat stress: Clinical implications and hormone treatments for the fertility of dairy cows. Theriogenology. 84(5), 659-666.

Dikmen, S., & Hansen. P. J. (2009). Is the temperature-humidity index the best indicator of heat stress in lactating dairy cows in a subtropical environment? J. Dairy Sci. 92(1), 109-116.

Ferreira, F. C., Gennari, R. S., Dahl, G. E., De Vries, A. (2016). Economic feasibility of cooling dry cows across the United States. J Dairy Sci. 99(12), 9931-9941. doi: 10.3168/jds.2016-11566.

Gao, S. T., Guo, J., Quan, S. Y., Nan, X. M., Fernandez, M. V. S., Baumgard, L. H., Bu. D. P. (2017). The effects of heat stress on protein metabolism in lactating Holstein cows, J Dairy Sci. 100, 5040–5049. <https://doi.org/10.3168/jds.2016-11913>.

Garner, J. B., Douglas, M., Williams, S. R. O., Wales, W. J., Marett, L. C., DIgiacomo, K., Leury, B. J., Hayes, B. J. (2017). Responses of dairy cows to short-term heat stress in controlled-climate chambers. Anim. Prod. Sci. 57, 1233-1241.

Gaughan, J. B., Davis, M. S., Mader. T. L. (2004).Wetting and the physiological responses of grain-fed cattle in a heated environment. Aust. J. Agric. Res. 55, 253-260.

Godyń, D., Herbut, P., Angrecka, S. (2019). Measurements of peripheral and deep body temperature in cattle – a review. J Therm Biol. 79, 42–49. doi: 10.1016/j.jtherbio.2018.11.011.

Gonzalez-Rivas, P. A., DiGiacomo, K., Russo, V. M., Leury, B. J., Cottrell, J. J., Dunshea. F. R. (2016). Feeding slowly fermentable grains has the potential to ameliorate heat stress in grain-fed wethers, J Anim. Sci. 94, 2981–2991. doi:10.2527/jas2016-0295.

Habeeb, A. A., Gad, A. E., Atta., M. A. (2018). Temperature-humidity indices as indicators to heat stress of climatic conditions with relation to production and reproduction of farm animals. Int J Biotechnol Recent Adv. 1(1), 35-50. doi: 10.18689/ijbr-1000107.

Hamzaoui, S., Salama, A. A. K., Albanell, E., Such, X., Caja, G. (2013). Physiological responses and lactational performances of late‐lactation dairy goats under heat stress conditions. J. Dairy Sci. 96(10), 6355‐6365. doi: 10.3168/jds.2013‐6665.

Havlík, P., Valin, H., Mosnier, A., Obersteiner, M., Baker, J. S., Herrero, M., Rufino, M. C., Schmid, E. (2013). Crop productivity and the global livestock sector: Implications for land use change and greenhouse gas emissions. Am. J. Agric. Econ. 95, 442–448.

Hoffmann, G., Schmidt, M., Ammon, C., Rose-Meierhöfer, S., Burfeind, O., Heuwieser, W., Berg. W. (2013). Monitoring the body temperature of cows and calves using video recordings from an infrared thermography camera. Vet. Res. Commun. 37(2), 91-99.

Holt. S. M., Gaughan, J. B., Mader. T. L. (2004). Feeding strategies for grain-fed cattle in a hot environment, Aust. J. Agric. Res. 55, 719-725. doi: 10.1071/AR03261.

Kadzere, C., Murphy, M., Silanikove, N., Maltz, E. (2002). Heat stress in lactating dairy cows: a review. Livestock Prod Sci. 77, 59–91. doi: 10.1016/S0301-6226(01)00330-X 9.

Kassube, K. R., Kaufman, J. D., Pohler, K. G., McFadden, J. W., Ríus., A. G. (2017). Jugular-infused methionine, lysine and branched-chain amino acids does not improve milk production in Holstein cows experiencing heat stress. Animal. 11, 2220–2228.

Lamp, O., Derno, M., Otten, W., Mielenz, M., Nürnberg, G., Kuhla. B. (2015). metabolic heat stress adaption in transition cows: differences in macronutrient oxidation between late-gestating and early-lactating German Holstein dairy cows. PLoS ONE. 10, e0125264. doi: 10.1371/journal.pone.0125264.

LCI. (1970). Patterns of transit losses. Livestock Conservation. Inc., Omaha, NE.

Lee, J. S., Kacem, N., Kim, W. S., Peng, D. Q., Kim, Y. J., Joung, Y. G., Lee, C., Lee. H. G. (2019). Effect of Saccharomyces boulardii supplementation on performance and physiological traits of holstein calves under heat stress conditions. Animals. 9, 510. doi:10.3390/ani9080510.

LPHSI. (1990). Livestock and Poultry Heat Stress Indices. Agricultural Engineering Technology Guide, Clemson University, Clemson, Sc. 29634, USA.

Marai, I. F-M., El-Darawanya, A. A., Fadiel, A., Abdel-Hafez, M. A. M. (2007). Physiological traits as affected by heat stress in sheep—A review. Small Rumin. Res. 71, 1-12.

Marai, I. F. M., Ayyat, M. S., El-Monem, U. M. (2001). Growth performance and reproductive traits at first parity of New Zealand White female rabbits as affected by heat stress and its alleviation under Egyptian conditions. Trop. Anim. Health Prod. 33(6), 451-462.

Mehaba, N., Salama, A. A. K., Such, X., Albanell, E., Caja, G. (2019). Lactational responses of heat-stressed dairy goats to dietary L-carnitine supplementation, Animals. 9, 567. doi:10.3390/ani9080567.

Opgenorth, J., Abuajamieh, M., Horst, E. A., Kvidera, S. K., Johnson, J. S., Mayorga, E. J., Sanz-Fernandez, M. V., Al-Qaisi, M. A., DeFrain, J. M., Kleinschmit, D. H., Gorden, P. J., Baumgard, L. H. (2021). The effects of zinc amino acid complex on biomarkers of gut integrity, inflammation, and metabolism in heat-stressed ruminants. J Dairy Sci. 104, 2410-2421. doi: 10.3168/jds.2020-18909.

Ortiz, X. A., Smith, J. F., Rojano, F., Choi, C. Y., Bruer, J., Steele, T., Schuring, N., Allen, J., Collier. R. J. (2015). Evaluation of conductive cooling of lactating dairy cows under controlled environmental conditions, J Dairy Sci. 98, 1759–1771 doi: 10.3168/jds.2014-8583.

Pinto, S., Hoffmann, G., Ammon, C., Amon. T. (2020). Critical THI thresholds based on the physiological parameters of lactating dairy cows. J Therm Biol. 88, 102523. doi: 10.1016/j.jtherbio.2020.102523.

Renaudeau, D., Collin, A., Yahav, S., De Basilio, V., Gourdine, J. L., Collier. R. J. (2012). Adaptation to hot climate and strategies to alleviate heat stress in livestock production. Animal. 6 (05), 707–728.

Rhoads, R. P., Baumgard, L. H., Suagee, J. K., Sanders, S. R. (2013). Nutritional interventions to alleviate the negative consequences of heat stress. Adv Nutrition. 4, 267–76. doi: 10.3945/an.112.003376.

Rispoli, L. A., Edwards, J. L., Pohler, K. G., Russell, S., Somiari, R. I., Payton, R. R., Schrick, F. N. (2019). Heat-induced hyperthermia impacts the follicular fluid proteome of the periovulatory follicle in lactating dairy cows. PloS One. 14(12), e0227095.

Rivera-Ferre, M. G., López-i-Gelats, F., Howden, M., Smith, P., Morton, J. F., Herrero. M. (2016). Re-framing the climate change debate in the livestock sector: Mitigation and adaptation options: Mitigation and adaptation options in the livestock sector. Wiley Interdiscip. Rev. Clim. Chang. 7, 869–892.

Rojas-Downing, M. M., Nejadhashemi, A. P., Harrigan, T., Woznicki. S. A. (2017). Climate change and livestock: Impacts, adaptation, and mitigation. Clim Risk Manag. 16, 145-163. doi: 10.1016/j.crm.2017.02.001.

Salama, A. A. K., Caja G., Hamzaoui, S., Badaoui, A., Castro‐Costa, A., Facanha, D. A. E., Gilhermino, M. M., Bozzi, R. (2014). Different levels of response to heat stress in dairy goats. Small Rumin. Res. 121(1), 73‐79. DOI: 10.1016/j.smallrumres.2013.11.021.

Sejian, V., Bhatta, R., Gaughan, J. B., Dunshea, F. R., Lacetera, N. (2018). Adaptation of animals to heat stress. Animal. 12(s2), s431–s444. doi: 10.1017/S1751731118001945.

Sevi, A., & Caroprese, M. (2012). Impact of heat stress on milk production, immunity and udder health in sheep: A critical review. Small Rumin. Res. 107, 1– 7.

Sevi, A., Annicchiarico, G., Albenzio, M., Taibi, L., Muscio, A., Dell’Aquila, S. (2001). Effects of solar radiation and feeding time on behavior, immune response and production of lactating ewes under high ambient temperature. J Dairy Sci. 84, 629–640.

Silanikove, N. (2000). Effects of heat stress on the welfare of extensively managed domestic ruminants. Livest. Prod. Sci. 67, 1-18. doi: 10.1016/S0301-6226(00)00162-7.

Speakman, J. R., & Krol, E. (2010). Maximal heat dissipation capacity and hyperthermia risk: neglected key factors in the ecology of endotherms. J Animal Ecol. 96, 726–46. doi: 10.1111/j.1365-2656.2010.01689.x.

Thornton, P., Nelson, G., Mayberry, D., Herrero, M. (2022). Impacts of heat stress on global cattle production during the 21st century: a modelling study. Lancet Planet. Health. 6(3), e192-e201.

Unruh, E. M., Theurer, M. E., White, B. J., Larson, R. L., Drouillard, J. S., Schrag, N. (2017). Evaluation of infrared thermography as a diagnostic tool to predict heat stress events in feedlot cattle. Am. J. Vet. Res. 78(7), 771-777.

Welfare Quality®. (2009). Welfare Quality® assessment protocol for cattle. Welfare Quality® Consortium, Lelystad, Netherlands.

Zhang, Y. W., McCarl, B. A., Jones, J. P. (2017). An overview of mitigation and adaptation needs and strategies for the livestock sector. Climate. 5(4), 95.

Zimbelman, R. B., Rhoads, R. P., Rhoads M. L., Duff, G. C., Baumgard, L. H., Collier. R. J. (2009). A re-evaluation of the impact of temperature humidity index (THI) and black globe humidity index (BGHI) on milk production in high producing dairy cows. In Proceedings of the Southwest Nutrition Conference (ed. RJ Collier). 158-169.
